# Associations Between the Self-reported Happy Home Lives and Health of Canadian School-aged Children: An Exploratory Analysis with Stratification by Level of Relative Family Wealth

**DOI:** 10.34763/jmotherandchild.20212503SI.d-21-00015

**Published:** 2022-04-30

**Authors:** Pianarosa Emilie, Colleen M Davison

**Affiliations:** 1 Dalla Lana School of Public Health, University of Toronto, Toronto, Ontario Canada; 2 Department of Public Health Sciences, Queen’s University, Kingston, Ontario Canada

**Keywords:** adolescent health, home life, social determinants of health, happiness, HBSC, Canada

## Abstract

**Background:**

Connections between home life, level of family wealth, happiness and health are strong, yet these relationships are complex and for Canadian adolescents not well studied. The objective of this investigation was to explore associations between aspects of health and self-reported happy home life among Canadian adolescents aged 10–16 years and to determine if level of self-reported relative family wealth modified associations.

**Material and methods:**

This was a secondary analysis of Canadian data from the 2018 Health Behaviour in School-aged Children (HBSC) study (n=21,745). Theory drove the selection of 26 health-related HBSC variables. Bivariate analyses and calculation of adjusted odds ratios, considering level of self-reported relative family wealth in a stratified analysis, were undertaken.

**Results:**

Overall, proximal, micro-level factors were most strongly associated with reports of a happy home life, with distal, macro-level factors less strongly associated. Differences existed between the health and home-life associations for adolescents of different levels of self-reported relative family wealth indicating effect modification. Family support and levels of adolescent self-reported overall health and mental health were common factors that were strongly associated with reporting a happy home life.

**Conclusion:**

We believe happy home lives are central and critical for thriving youth and families. This was an exploratory analysis. Many of the factors and relationships in this study are potentially modifiable and represent important possible areas of future focus for adolescent and family health improvement.

## Introduction

The World Happiness Report orders countries by an overall happiness score and in 2020, Canada ranked 15th most happy out of 153 countries, with Finland, Iceland and Denmark in the top three spots [1]. Within the Canadian population, adolescents are the group who report being the most happy with life when compared to all other age groups. Indeed, 97.9% of Canadian youth age 12–17 report being happy or very happy with their life [2]. In the global literature, evidence is mixed about whether adolescence is a developmental period of greater or lesser happiness. In the United States, like Canada, for example, adolescents are among the happiest age groups [3], yet in Finland, happiness appears lower in at least early adolescence [4].

Happiness has many definitions in academic literature. Michael Argyle [5], one of the first formal happiness scholars, separates happiness into measures of positive and negative affect and satisfaction. Many modern scholars use happiness and subjective well-being, particularly in an emotional or psychosocial sense, as synonymous and interchangeable terms [6, 7]. Positive psychology is a branch of psychology that focuses on the positive aspects of human experience, emphasising strengths, well-being and happiness [8, 9, 10, 11]. Although positive psychologists have begun to study the links between positive life experiences and health only recently, self-reports of happiness have been associated with lower rates of depression [12] and internalising problems [13] as well as with longevity [6], quicker recovery from illness [6], asthma diagnosis [14] and metabolic control of type I diabetes [15] among other outcomes. As it has relatively recently been applied to adolescent health [16], concepts in positive psychology likely represent many new avenues for health promotion, with the application of existing theory to its study still in its infancy.

Linley and colleagues [17] suggest that in order to understand the mechanisms underlying happiness and its influence on other aspects of individuals and populations, there is a need to explore how positive psychology fits with other frameworks, including those in population health and adolescent development. Urie Bronfrenbrenner’s socioecological model of human development [18, 19] has been engaged with from a ‘resilience’ perspective [20] for instance, and could be considered similarly using a happiness or subjective well-being lens. It is also unclear how a personal sense of happiness might interact with individuals’ other determinants of health such as gender, age, social and physical environments, or income level.

Family-related factors such as level of parental support and positive family relationships are recognised as primary contributors to youth happiness and life satisfaction [21, 22]. Young people interact with their family members primarily in the context of their home [23] and it is in the context of the home that many people begin to develop personal identity and connect to immediate determinants of health such as food, shelter, and social support [23]. The home is a key social environment, an important determinant of health, which holds some of the strongest influences in Bronfrenbrenner’s conceptualised ‘microsystem’ or immediate environments [19]. Levels of family wealth or poverty also strongly correlate with many health outcomes [24]. Relationships between family wealth and self-reported happiness [25] and life satisfaction [26, 27] among adolescents have been demonstrated; however, the nature and consistency of this relationship is still being studied.

The interplay between home life, youth happiness and health outcomes is apparent, yet, though happiness itself is well defined and measurable [28, 29, 30] the Oxford Happiness Questionnaire (OHQ, a ‘happy home life’ and the relationship between this and adolescent health is not well studied.

Many questions have not been answered, including in the Canadian context. What do adolescents mean when they report that they are happy, or not happy, with their home life? What characterises a happy home life for Canadian adolescents? Does self-reported happy or unhappy home life associate with health? How do happy home lives relate to other known determinants of adolescent health such as gender, socioeconomic status or ethnocultural group? Is a ‘happy or unhappy home life’ a modifiable factor? And if so, which kinds of related interventions might best support adolescents?

### Theory-Driven Selection of Variables

For the purpose of this exploratory study, a socioecological Mandala of Health developed by Hancock and Perkins was used to guide selection of health-related variables [31]. This model was slightly modified as factors that were less relevant for school-aged children (e.g., work, sick care system) were removed. Variables were selected from the HBSC questionnaire to align with each of the seven spheres of the adapted Mandala of Health, including: individual sphere, family sphere, four spheres of individual and family intersections, and an all-encasing community sphere.

Individual factors relate to the body, mind and spirit. This internal sphere of health is widely recognised and it is commonly used in holistic health models [32, 33]. It includes aspects unique to oneself such as physical health, beliefs and values, awareness and control of feelings, and view of self [34]. Surrounding the internal sphere is the family sphere. Family is instrumental in the development of values, attitudes, and habits [13, 14]. Family-based health behaviors and family social relationships are also key to child and adolescent health. Next, we find the intersections of individual and familial factors, which, while unique to each individual, cannot exist independently of one’s familial environment. A report written by the Canadian Minister of National Health and Welfare (Marc Lalonde) in the 1970s conceptualises the intersectionality of these factors [35]. The aim of his report was to illuminate determinants of health that exist outside of medicine and healthcare. Hancock and Perkins built on Lalonde’s ideas to create the four additional spheres in their Mandala for Health areas that exist at intersections of individual and familial factors: (1) personal behaviour (e.g., engagement in risky health behaviours, physical fighting with others); (2) psycho-social economic environment (e.g., socio-economic status, social support system); (3) human biology (e.g., genetic makeup; sex, age); (4) physical environment (e.g., housing, schools and neighborhoods). Finally, the largest sphere in the Mandala is community, where values, standards, support systems and networks have an impact at an individual level. Using these definitions, questionnaire variables were mapped onto the edited model and examples for each area selected [Table 1].

**Table 1:** Health-related items selected to map onto the seven parts of the Mandala of Health

**Individual health factors**	
Body	Subjective health complaints
	Perception of own health
Mind	Depression
	Body image
Spirit	Spirituality scale

**Family health factors**	
	Number of adults in the home
	Presence of siblings in the home
	Living in a foster home
	Eating meals as a family
	Family support scale

**Health factors at individual-family intersections**	
Personal behavior	Bullying others
	Age of onset of risky behavior
	Physical fights
	Dating violence
	Use of electronic devices
Psychosocio-economic environment	Family affluence
	Food insecurity
	Peer support
Human biology	Gender
	Age
Physical environment	Bedroom to oneself
	Size of home
	How free time is spent

**Community health factors**	
Social capital scale	
Urban/rural comparison	
Participation in organised activities	

To summarise, this was an exploratory study that focused on two understudied areas. First, we aimed to determine whether different aspects of adolescent health are associated with self-reported happy home lives in Canadian school-aged children. We did this to begin to explore the potential relationships between subjective well-being as it relates to one’s home life, and different health concepts or components. These relationships might represent potential mechanisms of effect (in either direction) and/or correlated concepts that could be studied further in the future. Second, because we were aware of the existing known effect family affluence level has on health, we wanted to explore the potential modifying effects of different socioeconomic status levels on any associations between health and happy home life self-reports. Our hypotheses, given existing theoretical and empirical literature, were, first, that reports of happy home lives would correlate strongest with factors in the family sphere (since these are inherent in the ‘home’ context), as well as those factors in the individual sphere that related to mental health (since these might be similar to level of happiness or subjective well-being). Second, we hypothesised that there would be an interactive effect of family wealth level such that we would see differences in the strength or nature of the associations between health and happy home life self-report at higher and lower levels of subjective family wealth.

## Material and methods

### Data source

The HBSC questionnaire is a general health and health behaviour survey that uses a systematic, cluster-sampling approach to collect data from students in grades 6–10 in Canada (approximate ages 10–16 years; n=21,745) [23]. The HBSC uses a weighted sample by province, sex, and school grade to ensure the national representativeness of the dataset [23]. This secondary data analysis used data collected from the 2018 HBSC.

### Description of variables

#### Health-related factors

As outlined in Table 1, the edited Mandala of Health was used to select the health-related variables in the HBSC questionnaire. Appropriate cutoff points, driven by practical knowledge or distribution, were chosen to code variables into bivariate distributions. In the case of risk-taking behavior, ‘early’ age was based on cutoff points established in the literature [23]: responses from those aged 13 or younger were considered as evidence of early risk-taking. Four scales were used (family support, peer support, spirituality, social capital) that had been previously established by research groups using HBSC data [23, 24, 36, 37]. Supplementary file 1 provides the details about each of the 26 health-related variables including the exact item wording, response options and details about coding or cut-point rationale.

#### Happy Home Life

Happy home life was measured as a single, subjective, self-reported question: ‘I have a happy home life’. Bivariate categories were created where reporting a happy home life was coded as 1 (strongly agree, agree), and not reporting a happy home life as 0 (neither agree nor disagree, disagree, strongly disagree). Similar subjective well-being measures are supported in recent literature, with reliability and validity found to be high across population groups [1].

#### Level of Relative Family Wealth

Self-reported, relative family wealth was measured using a single self-reported question: ‘How well off do you think your family is?’ An average or higher stratum (1 = very well off, quite well off, average) and a lower stratum (0 = not very well off, not at all well off) were coded. The use of subjective self-reported family income questions is controversial [38], however, previously established HBSC family affluence scales (FAS, FASII) are also problematic in a Canadian context since there have been low (α = 0.31) Cronbach’s α scores [39] and weak internal reliability in rural areas [39]. Complex SES scales are also prone to non-response bias, especially when children are required to disclose information about family income [38]. Previous Canadian studies have successfully used this single item as an indicator of subjective relative family wealth [39, 40].

#### Data analysis

Bivariate 2x2 contingency tables were constructed and unadjusted odds ratios (uOR) calculated to determine the odds of reporting a happy home life in specific subgroups compared to a selected referent group for each health-related variable. As this was an exploratory study and there were 26 different health-related variables, individual interaction terms for each variable (crossed with relative family wealth) or further modelling to account for all potential confounding factors in these associations were not conducted. However, given the likely critical nature of family income (level of poverty or wealth) adjusted common odds ratios (aOR) were calculated while controlling for self-reported, relative family wealth. This was the best variable for income that was available in the HBSC dataset. Stratum-specific odds ratios (ssOR) for lower and average or higher levels of relative family wealth were calculated to evaluate differences between the two stratums and the potential effect modification by relative family wealth. We recognise that numerous tests of association were undertaken. While no formal adjustment was used for multiple comparisons, caution was taken in evaluating statistical significance because of the potential for a significant finding to have occurred by chance. This study was reviewed and ethical approval granted by the Queen’s University Health Sciences Research Ethics Board (#6028267).

## Results

The study sample included 21,745 participants across grades 6–10 (Table 2).

**Table 2:** Demographic Characteristics of the 2018 Canadian Health Behaviour in School-aged Children Study (n= 21,745)

	n	(%)
Age		
≤11	2457	(11.5)
12	4207	(19.6)
13	4519	(21.1)
14	4619	(21.6)
≥15	5630	(26.3)
*Missing*	*313*	
Grade		
6-8	13079	(60.7)
9-10	8462	(39.3)
*Other*	*203*	
*Missing*	*1*	
Gender		
Male	10347	(48.0)
Female	10870	(50.4)
Neither term describes me	336	(1.6)
*Missing*	*192*	
Race		
White	14075	(66.1)
Black	767	(3.6)
Latin American	205	(1.0)
Indigenous (Metis, Inuit, First Nations)	1664	(7.8)
East & Southeast Asian	699	(3.3)
East Indian & South Asian	563	(2.6)
Arab & West Asian	299	(1.4)
Other (including mixed race)	3030	(14.2)
*Missing*	*443*	
Relative Family Wealth		
Well off	10028	(53.1)
Average	7174	(38.0)
Not well off	1697	(9.0)
*Missing*	*2846*	
Urban/rural status of municipality		
Rural area (< 1,000)	916	(4.2)
Small centre (1,000 — 29,999)	11244	(51.7)
Medium centre (30,000 – 99,999)	3787	(17.4)
Large urban centre (≥100,000)	5798	(26.7)
Adults in primary home		
Mother and Father	15080	(71.1)
Mother and Partner	1174	(5.5)
Mother only	3351	(15.8)
Father and Partner	255	(1.2)
Father only	647	(3.1)
Other	714	(3.4)
*Missing*	*524*	

*Values are unweighted*

Associations between the 26 health-related factors and self-reports of happy home lives are reported in Table 3 as they relate to the different parts of the Mandala of Health.

**Table 3:** Unadjusted, adjusted and stratum-specific odds of self-reporting a happy home life for the 26 health-related factors that map onto the Mandala of Health

	Happy Home Life								
	Bivariate			Adjusted†		Stratum Specific†			
						Lower Family Wealth*		Avg/Higher Family Wealth**	
Individual Factors	n	OR	(95% CI)	OR	(95% CI)	ssOR	(95% CI)	ssOR	(95% CI)
*Subjective Health Complaints*	21 745	-		-					
Often (daily/weekly)	4091	1.00		1.00		1.00	-	1.00	-
Rarely (monthly)	16 781	2.50	(2.33-2.70)	2.92	(2.70-3.16)	4.80	(3.83-6.06)	2.72	(2.49-2.96)
*Perception of Own Health*	21 745	-		-					
Poor/Fair	3 588	1.00		1.00		1.00	-	1.00	-
Good/Excellent	17 388	4.28	(3.99-4.59)	3.13	(2.89-3.40)	3.64	(2.90-4.57)	3.06	(2.81-3.34)
*Depression*	20 561	-		-					
Experiences symptoms	6 289	1.00		1.00		1.00	-	1.00	-
Does not experience	14 272	3.19	(3.00-3.41)	4.15	(3.86-4.46)	6.13	(4.93-7.63)	3.94	(3.65-4.26)
symptoms		-		-					
*Perception of Body Image*	21 745								
Too thin or too fat	8 654	1.00		1.00		1.00	-	1.00	-
About the right size	13 091	1.81	(1.70-1.92)	1.92	(1.79-2.06)	2.73	(2.22-3.35)	1.84	(1.71-1.98)
*Spirituality*	19 361			-					
Not or somewhat important	9 926	1.00		1.00		1.00	-	1.00	-
Important	9 435	1.33	(1.25-1.41)	2.08	(1.94-2.24)	2.97	(2.41-3.66)	1.99	(1.85-2.15)

	Happy Home Life								
	Bivariate			Adjusted†		Stratum Specific†			
						Lower Family Wealth*		Avg/Higher Family Wealth**	
Family Factors	n	OR	(95% CI)	OR	(95% CI)	ssOR	(95% CI)	ssOR	(95% CI)
*# of adults in the home*	21363								
1 adult	3 597	1.00	-	1.00	-	1.00	-	1.00	-
2 adults	15 789	2.06	(1.91-2.23)	2.00°	(1.86-2.22)	2.04°	(1.62-2.56)	2.03°	(1.85-2.23)
>2 adults	1 826	1.62	(1.43-1.83)	1.53	(1.33-1.75)	1.57	(1.02-2.41)	1.52	(1.32-1.76)
*Presence of siblings*	21 746								
No siblings	4 100	1.00	-	1.00	-	1.00	-	1.00	-
>1 sibling	1 168	1.24	(1.16-1.32)	1.22	(1.14-1.31)	1.33	(1.09-1.63)	1.20	(1.12-1.30)
*Foster home*	20 992								
Living in a foster home	20 752	1.00	-	1.00	-	1.00	-	1.00	-
Not in a foster home	239	1.69	(1.29-2.22)	1.71	(1.26-2.32)	0.68	(0.20-2.31)	1.83	(1.34-2.50)
*Eating meals as a family*	21 745								
<1 time a week	3 755	1.00	-	1.00	-	1.00	-	1.00	-
>1 time a week	17 860	2.77	(2.58-2.98)	2.90	(2.67-3.33)	4.11	(3.24-5.22)	2.76	(2.53-3.01)
*Family support scale*	21 745								
Family is not supportive	4 600	1.00	-	1.00	-	1.00	-	1.00	-
Family is supportive	15 675	3.62	(3.38-3.88)	4.76	(4.41-5.13)	5.35	(4.27-6.67)	4.69	(4.33-5.08)

	Happy Home Life								
	Bivariate			Adjusted†		Stratum Specific†			
						Lower Family Wealth*		Avg/Higher Family Wealth**	
Intersecting Factors	n	OR	(95% CI)	OR	(95% CI)	ssOR	(95% CI)	ssOR	(95% CI)
*Bullying*	21 745								
Has bullied others (past 3mo)	10 817	1.00	_-_	1.00	_-_	1.00	_-_	1.00	_-_
Has not bullied others (past 3mo)	10 928	2.17	(2.04-2.33)	1.64	(1.52-1.75)	1.61	(1.33-1.96)	1.64	(1.52-1.75)
*Risky behavior*	7 255								
< 12 years old	4 100	1.00	-	1.00		1.00	-	1.00	
> 12 years old	1 168	1.16	(1.07-1.26)	1.44	(1.32-1.57)	2.30	(1.76-3.00)	1.36	(1.24-1.49)
*Physical fights*	21 745								
In a physical fight (past 3mo)	14 521	1.00	_-_	1.00	_-_	1.00	_-_	1.00	_-_
Not in a physical fight (past 3 mo)	7 224	2.38	(2.22-2.50)	1.60°	(1.49-1.72)	1.43°	(1.15-1.75)	1.59°	(1.45-1.69)
*Dating violence*	7 655								
Has been violent with a partner	7 131	1.00	_-_	1.00	_-_	1.00	_-_	1.00	_-_
Has never been violent with a partner	524	1.07	(1.01-1.14)	1.28	(1.37-1.19)	1.89	(1.52-2.33)	1.23	(1.14-1.32)
*Electronic devices*	18 168								
Problematic use	1 945	1.00	_-_	1.00	_-_	1.00	_-_	1.00	_-_
Non-problematic use	16 223	2.08	(1.92-2.33)	2.63	(2.33-2.86)	2.22	(1.64-2.94)	2.63	(2.38-2.94)
*Food insecurity*	21745								
Food insecure	682	1.00	_-_	1.00	_-_	1.00	_-_	1.00	_-_
Not food insecure	20 970	3.57	(3.03-4.16)	3.46	(2.91-4.12)	1.95	(1.33-2.86)	3.97	(3.28-4.81)
*Peer support*	21745								
Peers are not supportive	5 003	1.00	_-_	1.00		1.00	-	1.00	-
Peers are supportive	15 290	1.39	(1.30-1.49)	1.75	(1.62-1.89)	1.46	(1.18-1.82)	1.79	(1.65-1.94)
*Family affluence*	19 054								
Not well off	10 715	1.00	_-_	1.00	_-_	_-_	_-_	_-_	_-_
Average/well off	8 340	3.22	(2.90-3.58)	3.22	(2.90-3.58)	-	-	_-_	
*Gender*	21745								
Female	11 162	1.00	_-_	1.00	_-_	1.00	_-_	1.00	_-_
Male	10117	1.39	(1.30-1.47)	1.50	(1.40-1.61)	1.62	(1.32-1.98)	1.49	(1.38-1.61)

	Happy Home Life								
	Bivariate			Adjusted†		Stratum Specific†			
						Lower Family Wealth*		Avg/Higher Family Wealth**	
Intersecting Factors	n	OR	(95% CI)	OR	(95% CI)	ssOR	(95% CI)	ssOR	(95% CI)
***Age***	21 745								
Grades 8-11	8 622	1.00	_-_	1.00	_-_	1.00	_-_	1.00	_-_
Grades 5-7	13 122	1.28	(1.20-1.37)	1.56	(1.45-1.67)	1.96	(1.59-2.43)	1.52	(1.39-1.64)
*Bedroom to oneself*	19 481								
Does not have a bedroom to oneself	2 467	1.00	_-_	1.00	_-_	1.00	_-_	1.00	_-_
Has a bedroom to oneself	17 014	0.98	(0.89-1.08)+	1.18	(1.06-1.30)	1.11	(0.85-1.45)	1.19	(1.06-1.32)
*Size of home*	19 499								
Small (<2 bathrooms)	13 043	1.00	_-_	1.00	_-_	1.00	_-_	1.00	_-_
Large (>2 bathrooms)	6 456	1.16	(0.77-1.79)+	1.23	(0.78-1.92)+	0.65	(0.22-1.96)	1.35	(1.00-2.22)
*Free time*	19 288								
No good places to spend free time	5 611	1.00	_-_	1.00	_-_	1.00	_-_	1.00	_-_
Good places to spend free time	13 617	2.46	(2.31-2.61)	1.75	(1.63-1.88)	1.63	(1.33-2.01)	1.76	(1.63-1.90)

	Happy Home Life								
	Bivariate			Adjusted†		Stratum Specific†			
						Lower Family Wealth*		Avg/Higher Family Wealth**	
Community Factors	n	OR	(95% CI)	OR	(95% CI)	ssOR	(95% CI)	ssOR	(95% CI)
*Social Capital Scale*	18 966								
Low social capital	17 821	1.00	-	1.00	-	1.00	_-_	1.00	-
High social capital	1 145	2.24	(2.08-2.40)	2.22	(2.09-2.42)	2.57	(2.05-3.22)	2.14	(1.97-2.32)
*Organized activities*	21 745								
Does not participate	2 749	1.00	-	1.00	-	1.00	-	1.00	-
Participates in ≥1 organized activity	18 996	1.25	(0.15-1.37)	1.44	(1.30-1.59)+	1.95	(1.50-2.51)	1.37	(1.23-1.52)
*Home location*	21 745								
Urban (>30,000 inhabitants)	11 931	1.00	-	1.00	-	1.00	-	1.00	-
Rural (<30,000 inhabitants)	9 814	1.08	(1.01-1.14)	1.05	(0.98-1.12)	1.03	(0.84-1.25)+	1.37	(1.23-1.52)

CI: confidence interval; OR: odds ratio; ssOR: stratum specific odds ratio. Note: Relative odds of reporting a happy home life are statistically different between the sub-groups in each variable if the OR with its 95% CI for the comparison sub-group do not include 1 (the referent group). †Models stratified by relative family wealth using a self-reported subjective scale. Perceptions of family wealth range from 1=very well off to 5= not at all well off. *Individuals who perceive their family as not at all well off or not very well off were categorised as a lower level of family wealth. ** Individuals who perceive their family as average, quite well off or very well off were categorised as average or high level of family wealth. ° aOR value not included within the ssOR range due to stratum cutoff point selection.

**Figure 1:**
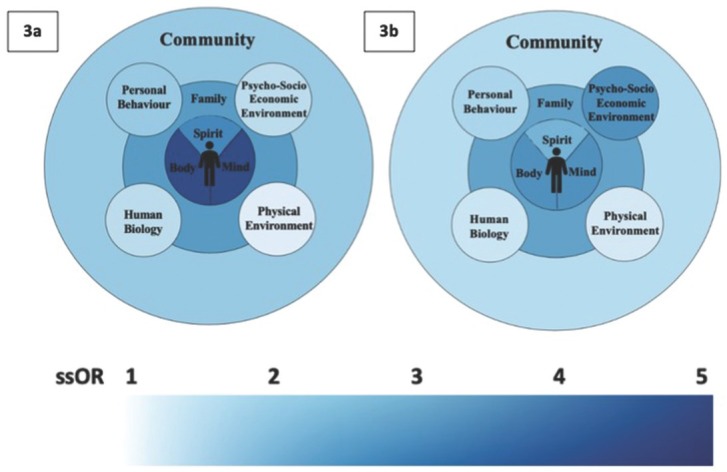
Adapted Mandala of Health shaded by mean stratum specific odds ratio for odds of the young person reporting a happy home life across the various health-related variables as compared to a referent group in each area of the model (a=lower self-reported family wealth; b= average or higher self-reported family wealth).

Unadjusted ORs are reported for each factor, along with the common aOR when controlling for subjective family wealth. Two strata – lower and average or higher self-reported relative family wealth – were evaluated. For the lower stratum, the odds of reporting a happy home life were greatest for individuals not experiencing symptoms of depression [ssOR = 6.13 (95% CI 4.93-7.63)], having strong family support [ssOR = 5.35 (95% CI 4.27-6.67)], students who rarely have subjective health complaints [ssOR = 4.80 (95% CI 3.87-6.06)] and those who reported eating meals as a family more than once a week [ssOR = 4.11 (95% CI 3.42-5.22)] compared to individuals who were not exposed to these factors. For the average or higher stratum, the odds of reporting a happy home life were greatest for individuals with strong family support [ssOR = 4.69 (95% CI 4.33-5.08)], not experiencing symptoms of depression [ssOR = 3.94 (95% CI 3.65-4.26)] and having a perception that their own health that is good or very good [ssOR = 3.06 (95% CI 2.81-3.34)] compared to those not exposed to these factors. The data indicates that there is effect modification by level of self-reported family wealth in most of the associations between health-related factors and self-reported happy home life. The ssOR 95% confidence intervals do not overlap for the two strata in the following factors: subjective health complaints, depression, body image, spirituality, eating meals as a family, partner violence and food insecurity. This indicates that the relationship between these health-related factors and self-reports of having a happy home life are different at different levels of self-reported family wealth (lower or average/higher). Figure 1 shows the adapted Mandala of Health for the lower (a) and average/higher (b) relative family wealth strata. Each section of the Mandala has been shaded according to the mean ssORs for that section. Thus, this highlights which types of health-related factors associate with self-reports of happy home lives and what the magnitude of these associates are. The lighter colour represents weaker association, while darker colour represents stronger association [Fig. 1].

## Discussion

Using data from 10–16 year old adolescents collected as part of the Canadian Health Behaviour in School-aged Children Study in 2018, we examined the association between specific health-related variables and self-reported happy home life. With the understanding that socio-economic situation is strongly linked to health outcomes and with some understanding that it plays a role in happy homes [21, 28], we explored the possibility of effect modification by relative family wealth. Overall, the direction and nature of the statistically significant associations are generally consistent with global happiness literature [1, 41, 42, 43, 44]. There were many strong associations and the magnitude of association between health-related variables and happy home lives appears to decrease as the spheres of influence move from micro-level (individual) factors to macro-level (community) factors in both of the family wealth strata. The World Happiness Report states that close personal relationships with loved adults (embodied by the family support scale in this study) explain the greatest variation in happiness [1]. When examining each independent variable in our associations, we see that the family support scale shows the strongest association with reports of a happy home life for average or higher family wealth strata, and the second strongest association for lower self-reported family wealth strata. This was in line with our initial hypothesis that family-related factors would most strongly associate.

Adolescents in the lower socioeconomic stratum appear to have a higher magnitude of association between many health-related variables and self-reports of happy home life as compared to individuals in the higher socioeconomic stratum. This is interesting, and although in line with our original hypothesis, does represent novel findings. For example, the magnitude of overall effect and the magnitude of the effect modification by relative family wealth is especially striking for food insecurity and depression. Meaning, the difference in the health and happy home life association is greatest between the two wealth strata for these variables. The odds of reporting a happy home life was greater for food secure individuals in our own study as compared to their food insecure counterparts and the magnitude of these odds is larger in students reporting average or higher family wealth [ssOR = 3.97 (95%CI 3.28-4.81)] compared to lower [ssOR = 1.95 (95% CI 1.33-2.86)]. What are the particular characteristics of home lives and of food insecurity/security experiences that create these differential effects? A qualitative Canadian study in Quebec presenting interview data for 98 households in rural and urban areas around Quebec City [45] concluded that shortage of food (present and possible in the future) is linked to experiences of socio-familial strain. It is possible that families in situations of average or higher relative family wealth would experience greater familial strain if they experienced food insecurity, as it might not be a common experience or one that they have yet adapted to. Another potential explanation for the difference in the associations across the two wealth strata might be related to the student’s different feelings of alienation or relative concern. Relative family wealth has been shown to be strongly associated with happiness [1] and anxiety [24], in making social comparisons, there might be a greater sense of alienation or concern felt by a relatively affluent individual who does not have their basic food needs met [45] than for someone less affluent.

The odds of reporting a happy home life are much greater for individuals who are not experiencing symptoms of depression as compared to those who are. This was in line with our original hypothesis, since mental health variables were predicted to most strongly associate with self-assessments of home life in both positive and negative directions. Positive Psychologists note that the strong overlap between feelings of happiness and mental well-being [5, 9]. The magnitude of these odds is intensified in the lower self-reported family wealth group [ssOR = 6.13 (95%CI 4.93-7.63)] as compared to the average or higher group [ssOR = 3.94 (95% CI 3.654.26)]. Other research groups have similarly found a significant association (p<0.001) between low socioeconomic status and depression [46]. Youth in higher socioeconomic classes experience less depressive symptoms overall [47] and this may be due to differential exposure to stressors and access to support services across social classes. There might also be differences in how aspects of home-life affect or interact with adolescent mental health across the two relative family wealth strata, and this warrants further exploration.

When comparing areas of the model across Figures 1a and 1b there are interesting differences. Most notably, individual-level factors (the inner sphere of the Mandala) have stronger associations with happy home lives for individuals in the lower self-reported family wealth stratum. We know that individuals at lower levels of affluence are at risk of not meeting their basic needs, making them more susceptible to physical, mental and emotional problems [45]. This could explain some of the differential odds that were found in this inner sphere especially.

Findings from the present study indicate that reports of a happy home life in adolescents are minimally associated with community factors, including social capital. This is inconsistent with literature showing strong associations between community factors and happy home lives among people of all ages [1] so would require further investigation. Differences may be due to the fact that our study focused on 10–16-year-old youth who may have a different kind of relationship to their community than older adults or younger children [7, 48].

### Strengths, limitations and future directions

The HBSC is a nationally representative survey that has a large sample size and is well pilot tested, making it ideal for use in secondary data analyses. The wide breadth of questions in the HBSC survey maps well onto the Mandala of Health and represent each selected sphere adequately. There are limitations to this study that arise largely from study sample selection. Participation bias may have been present due if the parental consent or youth assent to participate were differential or because of patterns of participant absence on the day the survey. Social desirability bias may exist because, although anonymous, youth are self-reporting and may overreport factors deemed positive and underreport factors deemed negative in an effort to be viewed favorably by others. The analysis method is subject to issues of multiple comparison (where the null hypothesis for independent tests is rejected although it is true [49]) due to the number of odds ratio calculations performed. Theoretically driven selection of included measures and the preparation of an analysis plan prior to performing odds ratios strengthens the studies’ interpretability. Nevertheless, significance should be interpreted with caution. Not all potential confounders are assessed for each association and it is likely that important confounders may have been overlooked for some associations. This serves as an exploratory study that will inform further investigations.

Future research could prioritise looking at the health factors that were most positively associated with self-reports of a happy home life. There are some common areas such as strong family support and lack of symptoms of depression that appear key for all adolescents. Additional qualitative or longitudinal quantitative data collection could be undertaken to further understand directions and mechanisms of effect and whether aspects are modifiable to better support adolescents and their families. The cross-sectional study approach does not allow assessment of causal pathways between health factors and happy home lives in either direction. A longitudinal cohort coupled with qualitative study would be ideal. Results from such a study could better inform future research and policy directions in support of the ongoing goal of improving adolescent and family health and happiness.

Health and happy home lives do appear closely linked for Canadian adolescents. Patterns of association between aspects of health and happy home lives differ by levels of subjective family wealth and this needs to be considered when designing intervention. This study highlights many potentially new avenues for further investigation and program development in efforts to support adolescent and family health.

### Key Points

- Self-reports of happy homes lives are associated with many health-related factors for Canadian adolescents aged 10–16 years.
- Many patterns of association between health factors and happy home lives differ by level of self-reported, relative family wealth.
- For adolescents who report lower levels of relative family wealth, self-reports of happy home life were most strongly associated with strong family support, lack of symptoms of depression, lack of general subjective health complaints, and families eating meals together. For adolescents who report average or higher levels of relative family wealth, happy home lives were associated with strong family support, lack of symptoms of depression, and positive perceptions of their own health.
- This study provides multiple directions for follow-up research, including exploring the direction and mechanisms of effect between happy home lives and health-related factors through longitudinal or qualitative research.
- Directly supporting parents so they can be strong supports for their adolescents at home as well as focusing on adolescent mental and self-perceived health appear to be key areas for continuing intervention focus.
- We believe happy home lives are central and critical for thriving youth and families. Many of the factors and relationships in this study are potentially modifiable, and this is an important area of future focus for adolescent and family health improvement.
