# Supplementary material for: Associations Between the Self-reported Happy Home Lives and Health of Canadian School-aged Children: An Exploratory Analysis with Stratification by Level of Relative Family Wealth
Source: J Mother Child. 2022 Apr 30;25(3):151–61. doi: 10.34763/jmotherandchild.20212503SI.d-21-00015 (PMC9097661; doi:10.34763/jmotherandchild.20212503SI.d-21-00015)
Supplement: Supplementary file 1 [file jmotherandchild-25-151_sm.pdf]

Supplementary File 1.

Specific item wording for the 26 health-related variables in the Happy Home Lives Study.

Individual health factors

– Subjective Health Complaints (headache, stomachache, backache, dizzy)

In the last 6 months, how often have you had the following? *(Please mark one box for each line)*

		About every day	*More than once a week*	*About every week*	*About every month*	*Rarely or never*
a.	Headache ***headache***	1☐	2☐	3☐	4☐	5☐
b.	Stomachache ***stomachache***	1☐	2☐	3☐	4☐	5☐
c.	Backache ***backache***	1☐	2☐	3☐	4☐	5☐
d.	Feeling low (depressed) ***feellow***	1☐	2☐	3☐	4☐	5☐
e.	Irritability or bad temper ***irritable***	1☐	2☐	3☐	4☐	5☐
f.	Feeling nervous ***nervous***	1☐	2☐	3☐	4☐	5☐
g.	Difficulties in getting to sleep ***sleepdificulty***	1☐	2☐	3☐	4☐	5☐
h.	Feeling dizzy ***dizzy***	1☐	2☐	3☐	4☐	5☐
i.	Sore mouth or toothache ***A46_9***	1☐	2☐	3☐	4☐	5☐

– Perception of own health

Would you say your health is ... ? ***health***

1☐ Excellent 2☐ Good 3☐ Fair 4☐ Poor

– Body Image

Do you think your body is ... ? ***thinkbody***

1☐ Much too thin

2☐ A bit too thin

3☐ About the right size

4☐ A bit too fat

5☐ Much too fat

– Depression

During the past 12 months, did you ever feel so sad or hopeless almost every day <u>for two weeks or more in a row</u> that you stopped doing some usual activities? ***A44***

1☐ Yes 2☐ No

– Spirituality scale

How important is it for you to … ? *(Please mark one box for each line)*

		Not at all important				*Very important*
a.	Be kind to other people. ***SPR1***	1☐	2☐	3☐	4☐	5☐
b.	Be forgiving of others. ***SPR2***	1☐	2☐	3☐	4☐	5☐
c.	Show respect for other people. ***SPR3***	1☐	2☐	3☐	4☐	5☐
d.	Feel that your life has meaning or purpose. ***SPR4***	1☐	2☐	3☐	4☐	5☐
e.	Experience joy in life. ***SPR5***	1☐	2☐	3☐	4☐	5☐
f.	Feel connected to nature or wilderness. ***SPR6***	1☐	2☐	3☐	4☐	5☐
g.	Care for the natural world. ***SPR7***	1☐	2☐	3☐	4☐	5☐
h.	Meditate or pray. ***SPR8***	1☐	2☐	3☐	4☐	5☐
i.	Feel a connection to a higher spiritual power. ***SPR9***	1☐	2☐	3☐	4☐	5☐
j.	Feel a sense of belonging to something greater than yourself. ***SPR10***	1☐	2☐	3☐	4☐	5☐

Family health factors:

– Number of adults in the home (motherhome, fatherhome, stepmohome1, stepfahome1, elsehome1)

Please answer this question for the home <u>where you live all or most of the time</u> and mark the box beside the people who live there.

1☐ Mother ***motherhome1***
2☐ Father ***fatherhome1***
3☐ Stepmother (or father's partner) ***stepmohome1***
4☐ Stepfather (or mother's partner) ***stepfahome1***
5☐ Brother or stepbrother *(How many?)* ***A7_7_______ A7_7_TEXT***
6☐ Sister or stepsister *(How many?)* ***A7_8________ A7_8_TEXT***
7☐ I live in a foster or children's home ***fosterhome1***
8☐ Someone else (e.g., grandparents). *Please write it down.* ***elsehome1***
.... ***A7_6_TEXT***
....

– Presence of siblings in the home (A7_7, A7_8)

Please answer this question for the home <u>where you live all or most of the time</u> and mark the box beside the people who live there.

1☐ Mother ***motherhome1***
2☐ Father ***fatherhome1***
3☐ Stepmother (or father's partner) ***stepmohome1***
4☐ Stepfather (or mother's partner) ***stepfahome1***
5☐ Brother or stepbrother *(How many?)* ***A7_7_______ A7_7_TEXT***
6☐ Sister or stepsister *(How many?)* ***A7_8________ A7_8_TEXT***
7☐ I live in a foster or children's home ***fosterhome1***
8☐ Someone else (e.g., grandparents). *Please write it down.* ***elsehome1***
.... ***A7_6_TEXT***
....

– Living in a foster home (fosterhome1)

Please answer this question for the home <u>where you live all or most of the time</u> and mark the box beside the people who live there.

- 1☐ Mother ***motherhome1***
- 2☐ Father ***fatherhome1***
- 3☐ Stepmother (or father's partner) ***stepmohome1***
- 4☐ Stepfather (or mother's partner) ***stepfahome1***
- 5☐ Brother or stepbrother *(How many?)* ***A7_7_______ A7_7_TEXT***
- 6☐ Sister or stepsister *(How many?)* ***A7_8________ A7_8_TEXT***
- 7☐ I live in a foster or children's home ***fosterhome1***
- 8☐ Someone else (e.g., grandparents). *Please write it down.* ***elsehome1***
  .... ***A7_6_TEXT***
  ....

– Eating meals as a family

How often do you and your family usually have meals together (breakfast, lunch, or dinner)? ***fmeal***

- 1☐ Every day
- 2☐ Most days
- 3☐ About once a week
- 4☐ Less than once a week
- 5☐ Never

– Family support scale

Please show how much you agree or disagree with the following statements.
(Please mark one box for each line)

		Very strongly disagree	*2*	*3*	*4*	*5*	*6*	*Very strongly agree*
a.	My family really tries to help me. ***famhelp***	1☐	2☐	3☐	4☐	5☐	6☐	7☐
b.	I get the emotional help and support I need from my family. ***famsup***	1☐	2☐	3☐	4☐	5☐	6☐	7☐
c.	I can talk about my problems with my family. ***famtalk***	1☐	2☐	3☐	4☐	5☐	6☐	7☐
d.	My family is willing to help me make decisions. ***famdec***	1☐	2☐	3☐	4☐	5☐	6☐	7☐

Health factors at individual-family intersections:

Personal behavior

- Bullying others

How often have you taken part in bullying another student(s) at school in the past couple of months? *bulliedothers*

1☐ I have not bullied another student(s) at school in the past couple of months

2☐ It has happened once or twice

3☐ 2 or 3 times a month

4☐ About once a week

5☐ Several times a week

- Age of onset of risky behavior

At what age did you first do the following things? *(Please mark one box for each line)*
If there is something you have not done, choose the 'never' category.

		Never	*11 years old or less*	*12 years old*	*13 years old*	*14 years old*	*15 years old*	*16 years or older*
a.	Drink alcohol (more than a small amount) ***A83_1***	1☐	2☐	3☐	4☐	5☐	6☐	7☐
b.	Get drunk ***A83_2***	1☐	2☐	3☐	4☐	5☐	6☐	7☐
c.	Smoke a cigarette (more than a puff) ***A83_3***	1☐	2☐	3☐	4☐	5☐	6☐	7☐
d.	Use cannabis ***A83_4***	1☐	2☐	3☐	4☐	5☐	6☐	7☐

- Physical fights

During the past 12 months, how many times were you in a physical fight? *fight12m*

1☐ I have not been in a physical fight in the past 12 months

2☐ 1 time

3☐ 2 times

4☐ 3 times

5☐ 4 times or more

– Dating violence (90 d/e/f)

During the past 12 months, how many times did … *(Please mark one box for each line)*

		I did not date or go out with anyone during the past 12 months	*0 times*	*1 time*	*2 or 3 times*	*4 or 5 times*	*6 or more times*
a.	someone you were dating or going out with physically hurt you on purpose? ***A90_1***	1☐	2☐	3☐	4☐	5☐	6☐
b.	someone you were dating or going out with purposely try to control you or emotionally hurt you? ***A90_2***	1☐	2☐	3☐	4☐	5☐	6☐
c.	someone you were dating use social media to hurt, embarrass, or monitor you? ***A90_3***	1☐	2☐	3☐	4☐	5☐	6☐
d.	you physically hurt on purpose someone you were dating or going out with? ***A90_4***	1☐	2☐	3☐	4☐	5☐	6☐
e.	you purposely try to control or emotionally hurt someone you were dating or going out with? ***A90_5***	1☐	2☐	3☐	4☐	5☐	6☐
f.	you use social media to hurt, embarrass or monitor someone you were dating? ***A90_6***	1☐	2☐	3☐	4☐	5☐	6☐

– Use of electronic devices (71 e/f/i)

During the past year, have you … *(Please mark "no" or "yes" for each line)*

		No	*Yes*
a.	regularly found that you can't think of anything else but the moment you will be able to use social media again? ***emcsocmed1***	1☐	2☐
b.	regularly felt dissatisfied because you wanted to spend more time on social media? ***Emcsocmed2***	1☐	2☐
c.	often felt bad when you could not use social media? ***Emcsocmed3***	1☐	2☐
d.	tried to spend less time on social media, but failed? ***Emcsocmed4***	1☐	2☐
e.	regularly neglected other activities (e.g., hobbies, sports) because you wanted to use social media? ***Emcsocmed5***	1☐	2☐
f.	regularly had arguments with others because of your social media use? ***Emcsocmed6***	1☐	2☐
g.	regularly lied to your parents or friends about the amount of time you spend on social media? ***Emcsocmed7***	1☐	2☐
h.	often used social media to escape from negative feelings? ***Emcsocmed8***	1☐	2☐
i.	had serious conflict with your parents, brother(s) or sister(s) because of your social media use? ***Emcsocmed9***	1☐	2☐

Psychosocio-economic environment

- Family affluence

How well off do you think your family is? ***A92***

1☐ Very well off

2☐ Quite well off

3☐ Average

4☐ Not very well off

5☐ Not at all well off

- Food insecurity

Some young people go to school or to bed hungry because there is not enough food at home. How often does this happen to you? ***A13***

1☐ Always 2☐ Often 3☐ Sometimes 4☐ Never

- Peer support

Please show how much you agree or disagree with the following statements.
(Please mark one box for each line)

		Very strongly disagree	*2*	*3*	*4*	*5*	*6*	*Very strongly agree*
a.	My friends really try to help me. ***friendhelp***	1☐	2☐	3☐	4☐	5☐	6☐	7☐
b.	I can count on my friends when things go wrong. ***friendcounton***	1☐	2☐	3☐	4☐	5☐	6☐	7☐
c.	I can share both my happy feelings and my sad feelings with my friends. ***friendshare***	1☐	2☐	3☐	4☐	5☐	6☐	7☐
d.	I can talk about my problems with my friends. ***friendtalk***	1☐	2☐	3☐	4☐	5☐	6☐	7☐

Human biology

- Sex or Gender

sex **Are you male or female?**

1☐ Male

2☐ Female

3☐ Neither term describes me

– Age

grade **What grade are you in?**

1☐	2☐	3☐	4☐
Grade 8	Grade 9	Grade 10	Grade 11

Physical environment

– Bedroom to oneself

Do you have your own bedroom for yourself? ***fasbedroom***

1☐ No 2☐ Yes

– Size of home

How many bathrooms (room with a bath/shower or both) are in your home? ***fasbathroom***

1☐ None 2☐ One 3☐ Two 4☐ More than two

– How free time is spent

How many hours a day, in your free time, do you usually spend … ?
(Please mark one box for ***weekdays*** *and one box for* ***weekend*** *for each item)*

			None at all	*About half an hour a day*	*About 1 hour a day*	*About 2 hours a day*	*About or more hours a day*
a.	Watching TV, videos (including YouTube or similar services), DVDs and other entertainment on a screen?	***Weekdays A72_1***	1☐	2☐	3☐	4☐	5☐
		Weekend A72_1WE	1☐	2☐	3☐	4☐	5☐
b.	Playing games on a computer, games console, tablet (like iPad), smartphone or other electronic device (not including moving or fitness games)?	***Weekdays A72_2***	1☐	2☐	3☐	4☐	5☐
		Weekend A72_2WE	1☐	2☐	3☐	4☐	5☐
c.	Using electronic devices such as computers, tablets (like iPad) or smartphones for other purposes (e.g., homework, emailing, tweeting, Facebook, chatting, surfing the internet)?	***Weekdays A72_3***	1☐	2☐	3☐	4☐	5☐
		Weekend A72_3WE	1☐	2☐	3☐	4☐	5☐

Community health factors:

- Social capital scale

Please say how you feel about these statements about the area where you live. *(Please mark one box for each line)*

		Strongly agree	*Agree*	*Neither agree nor disagree*	*Disagree*	*Strongly disagree*
a.	People say hello and often stop to talk to each other in the street. ***nb3***	1☐	2☐	3☐	4☐	5☐
b.	It is safe for younger children to play outside during the day. ***nb4***	1☐	2☐	3☐	4☐	5☐
c.	You can trust people around here. ***nb5***	1☐	2☐	3☐	4☐	5☐
d.	There are good places to spend your free time (e.g., leisure centres, parks, shops). ***nb6***	1☐	2☐	3☐	4☐	5☐
e.	I could ask for help or a favour from neighbours. ***nb7***	1☐	2☐	3☐	4☐	5☐
f.	Most people around here would try to take advantage of you if they got the chance. ***nb8***	1☐	2☐	3☐	4☐	5☐

- Urban/rural comparison

Linked through the Canadian census data, population size per census subdivision.

- Participation in organised activities

Are you involved in any of these kinds of organized activities or groups?
(Mark "yes" or "no" for each line)

		Yes	*No*
a.	Organized team sports (e.g., hockey, soccer, basketball) ***LS1***	1☐	2☐
b.	Organized individual sports (e.g., tennis, swimming, skating) ***LS2***	1☐	2☐
c.	Volunteer work ***A34_3***	1☐	2☐
d.	Arts groups (e.g., music, dance, drama) ***A34_4***	1☐	2☐
e.	Community groups (e.g., Scouts, Girl Guides, 4-H, cadets) ***LS5***	1☐	2☐
f.	Church or other religious/spiritual group ***LS6***	1☐	2☐
g.	Other activities or groups (e.g., chess, math, debate) ***A34_7***	1☐	2☐
